# Supplementary material for: Copepod life history evolution under high‐ and low‐food regimes
Source: Evol Appl. 2023 Jul 3;16(7):1274–83. doi: 10.1111/eva.13563 (PMC10363812; doi:10.1111/eva.13563)
Supplement: Supplementary file 1 — Figure S1: [file EVA-16-1274-s002.docx]

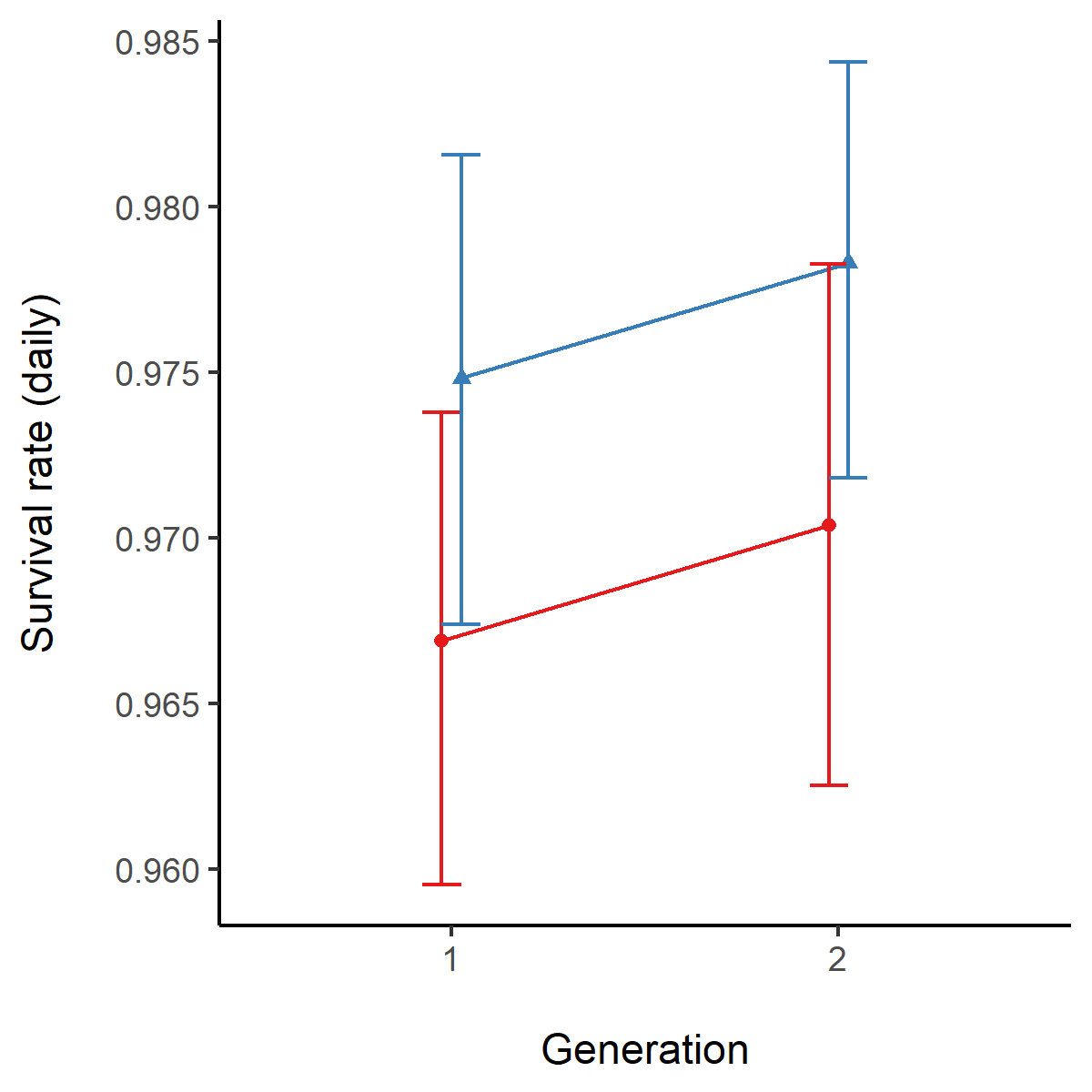


**Figure S1:** Mean daily survival rate, evaluated between hatching to 2 weeks after first observation of sexual maturity (within each replicate) in high-food (solid lines, circular points) and low-food (dotted lines, triangular points) lineages during G1 and G2 of common gardening. Error bars show between-culture bootstrapped 95% confidence intervals.
